# Improving cervical cancer screening rates among women of reproductive age in rural Kisumu County through dialogue-based community health education

**DOI:** 10.3332/ecancer.2024.1713

**Published:** 2024-06-13

**Authors:** Ochomo Edwin Onyango, David Masinde, Collins Ouma

**Affiliations:** School of Public Health and Community Development, Maseno University, Private Bag 40105, Maseno, Kenya

**Keywords:** cervical cancer, community, dialogue-based, health education, rural, screening

## Abstract

**Background:**

Low- and middle-income countries continue to bear the burden of cervical cancer partly due to low uptake of screening services. Interventions through the media to increase demand for screening services among women of reproductive age (WRA) have not yielded the desired results mainly due to the unidirectional flow of information. The current study evaluated the use of a dialogue-based approach to community health education to improve the demand for cervical cancer screening services among WRA in rural sub-counties in Kisumu County.

**Methods:**

This was a mixed-method longitudinal pre and post-intervention study with a control group. The self-reported screening rates were assessed at baseline in both the intervention and control groups followed by dialogue-based community health education in the intervention arm. This was followed by endline screening rates evaluation. The screening rates at baseline and endline were compared followed by a focused group discussion among the leaders of the community units to discuss the contributors to the observed screening rates. The proportion of change in the screening rates was calculated and statistical significance was assessed at *p* ≤ 0.05.

**Results:**

There was a significant increase in the number of WRA reporting to have been screened at the endline in the intervention arm (*p* = 0.007). The number of those being screened due to the health talks conducted by the Community health volunteers also increased significantly at the endline (*p* = 0.036). The barriers included; not knowing where to get screened (*p* < 0.0001), violation of ones’ privacy (*p* < 0.0001), lack of spousal support (*p* < 0.0001), waiting time at the facility (*p* = 0.001), attitude of the health providers (*p* < 0.0001) and cost of transport to the facility (*p* < 0.0001).

**Conclusion:**

The use of dialogue-based community health education has the potential to improve the uptake of cervical cancer screening services and identify the additional barriers as experienced by the WRA targeted for screening.

## Background

The burden of cervical cancer continues to rise globally with the low-and middle-income countries bearing the greatest burden. The global burden currently stands at 604,127 cases with 341,831 deaths [1]. East Africa, Kenya included, has one of the highest burdens with 54,560 cases and 36,497 deaths reported in 2020 [2]. In Kenya, cervical cancer is the second most frequent cancer in women of reproductive age (WRA), 15–49 years, where 5,236 cases and 3,211 deaths were recorded in 2020 [3].

The World Health Organisation recommends that 70% of women should be screened with a high-performance test by 35 years of age and again by 45 years of age and 90% of women identified with cervical disease should receive treatment at precancerous and those with invasive disease managed [4]. It has been shown that screening, early diagnosis and treatment of precancerous lesions prevent up to 80% of cervical cancer deaths [5]. Despite these ambitious targets and the possibility of positive treatment outcomes, the low- and middle-income countries still have low screening rates with an estimated 95% of the women having not been screened ever [5]. This low screening rate can be attributed to a lack of awareness in the population [5].

In Kenya, the screening rate remains between 14% and 16.4% for eligible women aged 18–69 years [6–8], and 16%–18% for women aged 25–49 years [9]. In Kisumu County, where the study was conducted, facility-based reports from Kenya Health Information System (KHIS) 2019 indicate that only 5.7% of the eligible women were screened for cervical cancer in 2019 and only 29,130 were screened in 2020 using the various methods which include human papillomavirus (HPV) testing, visual inspection using Lugol's iodine (VILI), visual inspection using acetic acid (VIA) and HPV.

The Ministry of Health currently uses audio-visual and print media to create awareness about cervical cancer screening services. However, this has not achieved the desired results in terms of cervical cancer screening rates, due to its inability to address specific questions held by the WRA targeted for screening. Community health volunteers (CHVs**)** have been shown to improve other health indicators such as immunisation coverage, deworming and antenatal clinic attendance if they have the right information. We evaluated the use of trained CHVs to create demand for cervical cancer screening services among WRA in the community by using a dialogue-based health education approach. The CHVs were trained on the dialogue model of community sensitisation and health education to understand the barriers and concerns of the community and relay the same feedback to the health facilities.

## Methods

### Study area

The study was carried out in the rural sub-counties of Kisumu County that is Nyando (Intervention group) and Nyakach Sub-Counties (Control group). Nyando Sub-County covers an area of 413.20 square kilometres with an estimated population of 161,508 of which 40,468 are WRA while Nyakach Sub-County covers an area of 357.30 square kilometres with an estimated population of 150,320 of which 38,011 are WRA according to the 2019 Kenya national census [10].

The two Sub-Counties were used because of their low cervical cancer screening uptake rates despite having robust and functional community health units and their comparability in terms of their rural nature [11]. The CHVs are the first level of healthcare provision, linking the community to the health facilities and serving as the entry point to the healthcare system providing basic services such as malaria testing and deworming among other basic services.

### Study design

This study adopted a quasi-experimental design (before and after with a control group) in which data were collected before (baseline) and after (post) implementation of the intervention with a control group. The intervention was implemented for a period of 10 months in the health facilities in Nyando Sub-County and to control for other programs and interventions, that might be happening during the study period, Nyakach was used as the control site. In the intervention site, the CHVs were trained on a dialogue-based approach to creating awareness among WRA before being allowed to do community sensitisation. The CHVs visited the households for their routine visits, during which they also shared information about cervical cancer with the household members through a dialogue-based approach. In the control site, the traditional general health talks and routine home visits normally done by the CHVs as a standard of care continued, with no information on cervical cancer added.

### Target population

The study targeted the WRA (15–49 years) residing in Nyando and Nyakach Sub-Counties. According to the 2019 Kenya national census report, the two sub-counties had a population of 75,364 WRA.

The sample size for each arm was calculated using the following formula by Willan and Briggs [12]:


$$n_{i}=\{p_{1}(1-p_{1})+p_{2}(1-p2\mathrm{)\}}{\left(\frac{Z}{E}\right)}^{2}$$

where *n* = required sample size for each arm p_1_ and p_2_= current cervical cancer screening rate in the county (6% according to KHIS 2019 data)

*z* = the value from the standard normal distribution at 95% CI (*z* = 1.96 for 95%)

*e* = degree of accuracy (0.05).

Therefore,

*n* = {0.06(1-0.06)+0.06(1-0.06)}(1.96/0.05)^2^

= {0.06(0.94)+0.06(0.94)}(39.2)^2^

*n* = 174 participants in each arm +10% for none response *n* = 192 in each arm.

The sample size was, therefore, 384 WRA.

The sample of 384 was then distributed according to the individual facility’s catchment, based on the number of community units (CUs) attached to it (see Appendix).

### Data collection

From the CHVs’ household registers, (MOH 513) potential participants were systematically identified and enrolled. In the control arm, 18 WRA and 15 WRA in the intervention arm were systematically selected per CU to arrive at the required sample size. The sampling interval was calculated using the formula:

Sampling interval per CU = Number of households/required sample size per CU

where a household sampled does not have an eligible respondent or is unwilling to participate in the study, the next household in the list was sampled.

Data were collected using a semi-structured questionnaire, which apart from assessing the WRA’s knowledge on cervical cancer, also captured self-reported screening status. The screening uptake was assessed before and after the sensitisation intervention by the trained CHVs.

### Data analysis

Univariate analysis was used to analyse the demographic characteristics of the study participants. Frequencies, percentages and proportions were determined and presented in tables and graphs. The chi-square test was used to determine the difference in categories between the variables in the control and intervention arm. A *p* ≤ 0.05 was considered statistically significant.

## Results

The study enrolled 384 participants; however, five could not complete the end-line survey and thus were excluded from the final analysis. Of the five excluded, four had moved out of the study area and could not be reached while one died within the study period. Therefore, the final analysis includes 189 respondents from the control arm and 190 from the intervention arm. These respondents were higher than the calculated sample sizes of *N* = 174 per group.

### Demographic characteristics of the study respondents

There were no significant differences between the two groups except for the level of education (*p =* 0.002) (Table 1).

### Cervical cancer screening rates among WRA in rural Kisumu County

The respondents were asked if they had ever been screened for cervical cancer and for those who confirmed that they had been screened when they were screened. There was a significant improvement in the number of WRA reporting to have been screened at the endline compared to the baseline in the intervention arm (*p =* 0.007). The increase in the number of those screened in the control arm was, however, not significant (Table 2).

### Facilitators to cervical cancer screening uptake among WRA in rural Kisumu County

The respondents, who reported to have been screened, were asked their reasons for being screened both at baseline and at endline. The number of those being screened due to the health talks conducted by the CHVs significantly increased at endline (*p =* 0.036). Other facilitators did not change significantly (Table 3).

### Barriers to cervical cancer screening uptake among WRA in rural Kisumu County

The women were also asked about the barriers to being screened for cervical cancer both at baseline and endline. There was a significant reduction in the number of respondents listing a lack of awareness about screening (*p <* 0.0001) in the intervention arm. However, significantly more respondents listed the following as barriers to screening at endline; not knowing where to get screened (*p <* 0.0001), lack of awareness on cervical cancer (*p <* 0.0001), fear of embarrassment (*p =* 0.027), not feeling at risk (*p <* 0.0001), violation of ones’ privacy (*p <* 0.0001), lack of spousal support (*p <* 0.0001), waiting time at the facility (*p =* 0.001), attitude of the health providers (*p <* 0.0001) and cost of transport to the facility (*p <* 0.0001).

In the control arm, there was a significant increase in the number of respondents listing violation of one’s privacy (*p <* 0.0001), attitude of the health provider (0.047), cost of transport to the facility (*p =* 0.004) and male health provider (*p =* 0.024) as barriers to being screened at endline (Table 4).

### Community health assistants (CHAs) and CHVs perspective on the barriers to cervical cancer screening

To further understand the persistent low cervical cancer screening uptake rates, focused group discussions were held among the CHAs and the chairpersons of the CUs.

### Feedback on dialogue-based community health education

The participants reported a notable shift in their approach to weekly home visits that had now adopted the dialogue-based approach. This approach allows the household members to air their concerns about service delivery at the local health facilities and it has borne great success in addressing health concerns within the community. The WRA has shown a newfound willingness to engage in cervical cancer screening. This positive response is attributed to the fact that they feel heard and understood within the community. One participant explained this shift, stating:

*‘People have embraced it currently and some come here to ask when it is done. Even yesterday some came to the CHV desk’.*

### Challenges in referring WRA for cervical cancer screening

The participants highlighted a range of challenges and barriers associated with the referral process for cervical cancer screening, affecting both healthcare providers and the women targeted for screening. Among the healthcare providers, including CHVs, several key challenges were identified. One significant issue is the exclusion of CHVs from implementation programs, limiting their involvement in the screening process. Additionally, there was a lack of adequate motivation and a notable knowledge gap among these providers. As one participant noted ‘*But the challenge we have faced is that most studies of cervical cancer screening, CHVs have not been brought on board, not even the CHAs’.*

Furthermore, the participants raised concerns about the gender of healthcare providers. WRA had reservations about being screened by male health providers, which often deterred them from seeking the necessary screening:

*‘When you want to refer somebody, they ask whether they will be seen by a male or female doctor? If you tell them they will be seen by a male doctor, most of them shy away’.*

The participants also pointed out that there is a shortage of healthcare providers who specialise in cervical cancer screening, and the few available lack sufficient experience to handle such cases. This deficiency in expertise can lead to incorrect information and results being provided, potentially endangering the individuals being screened:

*‘…sometimes we have providers who are not experienced enough, you know cervical cancer screening needs somebody who is competent and has knowledge about cervical cancer because when you give somebody wrong information and results that person is killing you slowly’.*

*‘Secondly, at times CHVs are less appreciated as it has been said, we work so hard in fact some are in the field as we talk, we work hard 24 hours a day and we receive calls even late at night’.*

In the case of the women targeted for screening, the participants highlighted a number of challenges including long distances to the facilities since not all health facilities offer these services. As one participant explained.* ‘It’s rare for somebody to leave their work just to come for cancer screening at the hospital because of the distance and fare involved. For instance, one may see it too costly spending 200/- to the hospital just for screening but instead use the same amount to buy flour’.*

Another significant barrier is the fear associated with the screening procedure itself. This fear is often rooted in prior experiences of painful procedures, misconceptions surrounding cervical cancer screening, and reservations regarding male healthcare providers performing the screenings.

*‘So it also depends with the information given out by those who have undergone the procedure, some instil fear. Some give information that instil fear to those who have not undergone the procedure. They may say that they experienced pain from the insertion of the metallic substance and they may never do it again’.*

Another concern raised by the participants was the issue of privacy. Most women fear potential breaches of confidentiality by the health care providers following their screening. They worry that these providers would share their personal and screening information to third parties, thus opening doors for stigmatisation within the community. ‘*I know how the process is being done, you know when someone is intruding into your privacy, you know that cervix is about privacy, this is because someone wants to know which panty you are wearing, how down there (private part) is clean, you see when you want to go there you start asking yourself that is the person I am going to meet there will she or he be able to keep the secret after seeing how my cervix look like, will she keep it to herself what she has seen there, that is another fear’.*

*‘It is important to recognise that we have stigma in the community and it is very difficult for one to come to the facility thus if screening is done at the community level, we will get majority’.*

### Suggested areas of improvement

The participants suggested valuable insights into how the government can enhance cervical cancer screening uptake among WRA in the community. These suggestions encompass several key areas

- The government should undertake community sensitisation outreach and dialogue days to create awareness on cervical cancer screening.
- Train more healthcare providers on cervical cancer screening services.
- Facilities should have health care providers specifically handling cervical cancer services to reduce time wastage waiting with other patients.
- The government should engage in school-based health talks and sensitisation on cervical cancer screening especially among teenage girls.
- The CHVs should be provided with means of transport, personal protective equipment and job aids to assist in their work.
- The government should work on increasing the remuneration of the CHVs to boost on their morale and improve on their livelihood.

*As one participant emphasized, ‘So we are requesting if you can a train many health care providers because this challenge, we are really experiencing it with CHA, mobilization, you will find that you have picked, trained and sensitize on cancer only one CHA in a facility, do you think the information will go back in the community and spread to the whole of the community? It will not, right?’*

*Additionally, another participant noted the importance of continuous training, saying, ‘For us to work well we need to be knowledgeable. This requires undergoing various trainings. A times training is done and then it takes so long even years before the next training and people tend to forget’.*

*‘Lastly, the suggestion for seeking external partners to support their initiatives was highlighted’*

*‘We also have secondary schools around with females of reproductive age. So, if we start school-based health talk some of the issues can be addressed in school’.*

*‘So you can find for us other partners to support us in this initiative like was started by this study. The income is very little and not steady and if we can get a partner who can raise it a little, we will appreciate’.*

## Discussion

The current study reported low cervical cancer screening rates among the study participants in both arms. Similar low screening rates were reported by a study among women in Kenya where only 35.6% of the eligible women were screened [13]. Among Ghanaian women, only 24.6% of those eligible were screened despite the close proximity to facilities offering free cervical cancer screening services [14]. This low screening rate is an indication of the existence of barriers to screening uptake, which remain unaddressed.

However, significant improvement was reported in the intervention arm following the dialogue-based sensitisation intervention which significantly improved the CHVs knowledge [15]. Similar results were also reported in a study among marginalised population of women in inner-city Durban, South Africa, those who reported having ever been screened were about a third, however, this increased to 64% following an educational session [16]. This was also in agreement with the findings of another systematic review and study among women living with HIV in Malawi which found educational interventions and community engagement to be effective in improving screening uptake [17, 18].

A systematic review identified CHWs to play an important role in cervical cancer screening mainly through community education, outreach and awareness activities [19]. Furthermore, receiving clear information on cervical cancer and access to mass campaign information were found to encourage cervical cancer screening uptake [20]. These results point to the potential of educational intervention and community engagement to address the barriers to screening uptake leading to increased demand for cervical cancer screening.

Despite the improvements in screening uptake following the educational intervention, still, a large proportion of the respondents reported not having been screened. This is an indication of the existence of barriers to screening, which have not been addressed. Some of the barriers listed by those not screened include; lack of knowledge, fear of the results and staff attitude. This is in agreement with a cross-sectional survey among women in Abidjan, Côte d'Ivoire, which reported the barriers to screening to include lack of sufficient knowledge on cervical cancer, carelessness or negligence, fear of cancer, fear of additional costs and the fear of a bad reception at the cervical cancer screening facility [20]. Another systematic review to determine the barriers to cervical cancer screening in low- and middle-income countries reported; lack of information about cervical cancer and its treatment, fear of embarrassment or shy, lack of time and lack of family support as the most commonly reported barriers [21]. A study by Petersen *et al* [22], identified a lack of knowledge and awareness of cervical cancer in general and of screening as the most frequently reported barrier to screening.

In a systematic review to explore ways to improve HPV vaccination and cervical cancer screening uptake in rural setups, it was noted that only a few interventions engage stakeholders to develop community-specific solutions to overcome obstacles associated with service delivery [23]. The respondents in the current study who lamented about being side-lined when interventions are being implemented also echoed similar sentiments.

The main facilitator to being screened was the recommendation of screening by a healthcare provider. A majority of those screened reported to have been referred by either the clinician or the CHVs who are part of the healthcare system. This finding is similar to the results of a systematic review which reported that being recommended by a healthcare worker to attend screening was a significant facilitator to getting screened [24]. Referral by the service providers is effective since it is accompanied by appropriate information required by the WRA to make a decision on screening since these healthcare providers have better knowledge of cervical cancer.

The other facilitators to cervical cancer screening uptake identified include; family or social support; positive attitude, motivation and perception of the benefit of screening and seriousness of cervical cancer; having health insurance and short travel distance to a facility offering screening service; women having higher education and socioeconomic status; counselling and support; ease of access; health promotion and advocacy; healthcare provider’s gender; and quality of screening services [25]. These are indications of how important a holistic approach to improving cervical cancer screening is important in ensuring that all the facilitators are enhanced while at the same time addressing the barriers.

## Limitation of the study

The study was limited by the source of data being participant-reported, which was affected by recall bias in the year of screening and the factors affecting screening decisions. Second, some participants could not differentiate between cervical cancer screening and general pelvic examination. Finally, this study was limited to only facilities that reported to be actively doing cervical cancer screening and thus may have limited generalisability.

## Conclusion

Cervical cancer screening rates among the WRA in Kisumu remain suboptimal; however, educational intervention by the trained CHVs was able to improve the screening rates significantly. Despite the improvement, lack of adequate information remains a critical barrier, which needs to be addressed to ensure more eligible women are screened. Finally, referral by the healthcare providers including the CHVs is an effective way to get these women to take up screening services.

## List of abbreviations

CHV, Community health volunteer; CU, Community health unit; KHIS, Kenya Health Information System; WRA, Women of reproductive age.

## Conflicts of interest

The authors declare that they have no competing interests.

## Funding

This study was not funded.

## Consent for publication

Not applicable.

## Ethics approval

This study was conducted in accordance with the Declaration of Helsinki. Scientific approval for this study was be obtained from Maseno University’s School of Graduate Studies (SGS) while ethical approval was obtained from Maseno University Ethics Review Committee (MUERC) (MUERC/00910/20). A research license was obtained from NACOSTI (Ref# 526448). The authority of the Kisumu County Health Management was also sought [Ref. GN 133 VOL VIII (473)]. Before recruitment into the study, the participants’ written informed consent was also obtained. Finally, the confidentially of the information and the identity of the participants was guaranteed by assigning unique identifiers to the participants. Access to data was limited to the principal investigator and the data were kept in locked cabinets and in folders protected with passwords to enhance confidentiality.

## Availability of data and materials

The raw data supporting the conclusions of this article will be made available by the authors, without undue reservation.

## Author contributions

OEO designed and carried out the data collection in the field and participated in the drafting of the manuscript. DM and CO made substantial contributions to the design and interpretation of the data. DM and CO were also involved in revising the manuscript critically for important intellectual content. They also gave the final approval of the version to be published and have agreed to be accountable for all aspects of this work. All authors read and approved the final manuscript.

## Scope statement

The manuscript presents new data on how the use of dialogue-based community health education improved the uptake of cervical cancer screening among WRA in rural sub-counties, Kisumu County. Low- and middle-income countries continue to bear the burden of cervical cancer partly due to low uptake of screening services. Interventions to increase demand for screening services among the WRA have not yielded the desired results mainly due to the unidirectional flow of information. The current study evaluated the use of dialogue-based approach to community health education to improve the demand for cervical cancer screening services among WRA in rural sub-counties in Kisumu County. We present data showing that there was a significant increase in the number of WRA reporting to have been screened at endline in the intervention arm (*p =* 0.007). The number of those being screened due to the health talks conducted by the CHVs also increased significantly at endline (*p =* 0.036). The results of this study highlight how the use of dialogue-based community health education has improved the uptake of cervical cancer screening services and identified additional barriers experienced by the WRA targeted for screening.

## Figures and Tables

**Table 1.** Demographic characteristics of the study respondents.

Variable	Categories	Intervention (*n* = 190)	Control (*n* = 189)	*p*-value
		*n* (%)	*n* (%)	
Age	18–24	39 (20.5)	32 (16.9)	0.843
	25–30	56 (29.5)	66 (34.9)	
	31–34	43 (22.6)	42 (22.2)	
	35–40	34 (17.9)	29 (15.3)	
	41–44	15 (7.9)	17 (9.0)	
	45–49	3 (1.6)	3 (1.6)	
Education level	No education	1 (0.5)	0 (0.0)	**0.002**
	Primary	56 (29.5)	39 (20.6)	
	Secondary	100 (52.6)	86 (45.5)	
	Tertiary	33 (17.4)	64 (33.9)	
Religion	Christian	188 (98.9)	184 (97.4)	0.326
	Muslim	2 (1.1)	3 (1.6)	
	No religion	0 (0.0)	2 (1.0)	
Marital status	Single	33 (17.4)	35 (18.5)	0.88
	Married	133 (70.0)	133 (70.4)	
	Divorced/widowed/separated	24 (12.6)	21 (11.1)	
Occupation^a^	Small scale farming	67 (35.3)	56 (29.6)	0.118
	Commercial farming	1 (0.5)	2 (1.0)	
	Business	76 (40.0)	80 (42.3)	
	Formal employment	12 (6.3)	26 (13.8)	
	Casual employment	20 (10.5)	12 (6.3)	
	Other	18 (9.5)	20 (10.6)	
HIV status	Positive	37 (19.5)	37 (19.6)	0.163
	Negative	147 (77.4)	151 (79.9)	
	Don’t know	6 (3.1)	1 (0.5)	

Data is in numbers (proportion).

a Multiple responses were allowed. Level of significance was determined at *p* ≤ 0.05. Bold are statistically significant.

**Table 2.** Screening rates among WRA in rural Kisumu County.

Variable	Intervention			Control		
	Baseline (*n* = 190)	Endline (*n* = 190)	*p*-value	Baseline (*n* = 189)	Endline (*n* = 189)	*p*-value
Ever screened						
No	138 (72.6)	113 (59.5)	0.007	136 (72.0)	132 (69.8)	0.651
Yes	52 (27.4)	77 (40.5)		53 (28.0)	57 (30.2)	
Duration since last screening						
<1 year	15 (28.9)	21 (27.3)	0.473	10 (18.9)	3 (5.3)	0.086
1–4 years	32 (61.5)	52 (67.5)		36 (67.9)	46 (80.7)	
5–9 years	5 (9.6)	3 (3.9)		7 (13.2)	8 (14.0)	
10–14 years	0	1 (1.3)		0	0	

Data are numbers (proportion). Denominator is the number of respondents. Statistical significance was reported at *p* ≤ 0.05. Bold is statistically significant.

**Table 3.** Facilitators to cervical cancer screening among WRA in rural Kisumu County.

Facilitators	Intervention			Control		
	Baseline (*n* = 52)	Endline (*n* = 77)	*p*-value	Baseline (*n* = 53)	Endline (*n* = 57)	*p*-value
Health talk by CHV	10 (19.2)	28 (36.4)	0.036	19 (35.9)	21 (36.8)	0.914
Advert from the media	8 (15.4)	12 (15.6)	0.975	1 (1.9)	1 (1.8)	0.959
Advice from health professionals	38 (73.1)	45 (58.4)	0.089	36 (67.9)	40 (70.2)	0.799
Advice from a friend or relative	5 (9.6)	10 (13.0)	0.558	2 (3.8)	2 (3.5)	0.941
Personal decision	1 (1.9)	1 (1.4)	0.639	0	0	Null
IUCD insertion	0	0	0.776	1 (1.9)	0	Null
Had infection in the private part	10 (19.2)	0	0.649	0	0	Null
Personal decision	8 (15.4)	1 (1.3)	0.688	1 (1.9)	1 (1.7)	0.639
Experienced pain in the genitals	38 (73.1)	1 (1.3)	0.649	0	0	Null

Data are numbers (proportion). Denominator is the number of respondents who reported to have been screened for cervical cancer. Statistical significance is reported at *p ≤ 0.05*. Null is where no observations were made across the two timelines. Bold is statistically significant.

**Table 4.** Barriers to cervical cancer screening among WRA in rural Kisumu County.

Variable	Intervention			Control		
	Baseline (*n* = 138)	Endline (*n* = 113)	*p*-value	Baseline (*n* = 136)	Endline (*n* = 132)	*p*-value
Barriers						
Lack of awareness about screening	89 (64.5)	19 (16.8)	<0.0001	69 (50.7)	68 (51.5)	0.898
Don't know where to get screened	14 (10.1)	31 (27.4)	<0.0001	28 (20.6)	29 (22.0)	0.782
Lack of awareness about cervical	23 (16.7)	44 (38.9)	<0.0001	28 (20.6)	33 (25.0)	0.389
Not feeling sick	3 (2.2)	8 (7.1)	0.059	12 (8.8)	16 (12.1)	0.378
Fear of embarrassment	9 (6.5)	17 (15.0)	0.027	26 (19.1)	31 (23.5)	0.382
Not feeling at risk	21 (15.2)	53 (46.9)	<0.0001	23 (16.9)	31 (23.5)	0.180
Violation of ones' privacy	7 (5.1)	26 (23.0)	<0.0001	3 (2.2)	18 (13.6)	<0.0001
Lack of spousal support	11 (8.0)	31 (27.4)	<0.0001	15 (11.0)	24 (18.2)	0.097
Societal stigma	8 (5.8)	11 (9.7)	0.241	9 (6.6)	10 (7.6)	0.760
Waiting time at the health facility	16 (11.6)	31 (27.4)	0.001	43 (31.6)	44 (33.3)	0.764
Attitude of the health providers	4 (2.9)	21 (18.6)	<0.0001	2 (1.5)	8 (6.1)	0.047
Cost of transport to the facility	12 (8.7)	37 (32.7)	<0.0001	9 (6.6)	24 (18.2)	0.004
Cultural beliefs	1 (0.7)	5 (4.4)	0.056	2 (1.5)	2 (1.5)	0.976
Male healthcare provider	27 (19.6)	32 (28.3)	0.104	3 (2.2)	11 (8.3)	0.024

Data are numbers (proportion). Denominator is the number of respondents who reported to have not been screened for cervical cancer. Statistical significance is reported at *p* ≤ 0.05. Bold is statistically significant.

## Appendix

**Table:**

S/No.	Health Facility	Sub-County	Arm	Number of CU.	Respondents
1	Bonde	Nyakach	Control	1	18
2	Pedo	Nyakach	Control	1	18
3	Onyuongo	Nyakach	Control	1	18
4	Radienya	Nyakach	Control	1	18
5	Sigoti	Nyakach	Control	2	34
6	Oboch	Nyakach	Control	1	18
7	Kibogo	Nyakach	Control	1	18
8	Kodingo	Nyakach	Control	3	50
9	Bunde	Nyando	Intervention	1	15
10	Magina	Nyando	Intervention	1	15
11	Koduol	Nyando	Intervention	1	15
12	Nyangande	Nyando	Intervention	3	44
13	HongoOgosa	Nyando	Intervention	2	29
14	Kochieng	Nyando	Intervention	1	15
15	Okana	Nyando	Intervention	1	15
16	Kadinda	Nyando	Intervention	1	15
17	Nyakongo	Nyando	Intervention	2	29
	Total	NA		24	384
